# The cytotoxic evaluation of mineral trioxide aggregate and bioaggregate in the subcutaneous connective tissue of rats

**DOI:** 10.4317/medoral.19095

**Published:** 2013-05-31

**Authors:** Yusuf B. Batur, Gözde Acar, Yagmur Yalcin, Seckin Dindar, Hande Sancakli, Ugur Erdemir

**Affiliations:** 1 PhD, DDS. Department of Endodontics, Faculty of Dentistry, Istanbul University (34093) Capa, Istanbul/Turkey; 2 DS. Department of Endodontics, Faculty of Dentistry, Istanbul University (34093) Capa, Istanbul/Turkey; 3 PhD, DDS. Department of Restorative Dentistry, Faculty of Dentistry, Istanbul University (34093) Capa, Istanbul/Turkey

## Abstract

Objectives: The purpose of this study was to evaluate and compare the cytotoxic effects of ProRoot MTA and DiaRoot BA, a bioceramic nanoparticulate cement, on subcutaneous rat tissue. Study Design: Fifty Sprouge Dawley rats were used in this study. Polyethylene tubes filled with ProRoot MTA and DiaRoot BioAggregate, along with a control group of empty, were implanted into dorsal connective tissue of rats for 7, 15, 30, 60, and 90 days. After estimated time intervals the rats were sacrificed. The specimens were fixed, stained with hematoxylin and eosin, and then evaluated under a light microscope for inflammatory reactions and mineralization. Results: All groups evoked a severe to moderate chronic inflammatory reaction at 7 and 15 days, which decreased with time. Both the MTA and BioAggregate groups showed similar inflammatory reactions, except at 90 days when MTA showed statistically significant greater inflammation (p>0.05). The MTA group showed foreign body reaction at all times. Compared to BioAggregate, MTA showed significantly more foreign body reaction at 60 and 90 days (p<0.0001). After 30 days foreign body reaction of BioAggregate decreased significantly. Both MTA and BioAggregate groups showed similar necrosis at 7 and 15 days (p=0.094 and p=0.186 respectively). No necrosis was observed after 15 days. Similarly there was no fibrosis after 30 days for both MTA and BioAggregate groups (p>0.05). Conclusions: Since DiaRoot BioAggregate showed significantly better results than MTA, we can conclude that it is more biocompatible. However, further studies are required to confirm this result.

** Key words:**Biocompatibility, mineral trioxide aggregate, bioAggregate.

## Introduction

Mineral trioxide aggregate (MTA) is the most frequently and preferentially used material in dental practice for sealing the communication between root canal system and periodontium. It is composed of tricalcium and dicalcium silicate, tricalcium aluminate, calcium sulfate (gypsum) (1), and a 4-to-1 addition of bismuth oxide for radiopacity. It is marketed as MTA ProRoot MTA (Dentsply, Tulsa Dental Products, Tulsa, OK) (2). Because of its remarkable biological and physical properties it is widely used in dental practice in contact with both soft and hard tissues as a root-end filling and apical barrier material, root canal perforation repair agent, pulp capping material, intraorifice barrier, and paste for root canal obturation material (3,4). MTA is very similar to Portland cement (1). Due to the materials used to manufacture Portland cement, it may contain some heavy metals including arsenic, chromium, and lead with amounts varying between 5 and 100 parts per million (ppm). Because of its similarity to Portland cement, there is some concern that MTA could also release hazardous substances (5). There is a lack of information about how MTA is manufactured. The manufacturer claims that the production is performed under isolated and clean conditions to eliminate the risk of contamination. The materials used to manufacture MTA are certified for being pure and free of heavy metal contamination (2). Despite their similarities, there are two very important differences between MTA and Portland cement. First, bismuth oxide is only found in MTA. Second, MTA has a lower tricalcium aluminate level. This implies that the two materials are not manufactured in the same way (5).

DiaRoot Bio-Aggregate (BA) (Innovative BioCaramix Inc, Vancouver, BC, Canada) is a new water-based cement. Like MTA, it is used for retrograde filling and root canal perforation repair. The composition of BA is tricalcium silicate, dicalcium silicate, tantalum pentoxide, and calcium phosphate monobasic. To provide radiopacity, tantalum pentoxide is used in BA rather than the bismuth oxide used in MTA. The manufacturer claims that DiaRoot is produced under controlled conditions to form contamination-free biocompatible ceramic nanoparticles (DiaRoot; DiaDent, Burnaby, BC, Canada). It is the first nanoparticulate repair cement introduced on the dental market. It is claimed that BA promotes cementogenesis and forms a hermetic seal; however, studies do not support this claim (3,6).

MTA and BA have similar compositions and uses. The most significant difference between these two products is that BA is alu-minum-free. This is important because irritation caused by toxic effects of such endodontic repair materials, which come into contact with both soft and hard tissues, may be responsible for degeneration of the periapical tissue and/or delayed wound healing (7). Because both cements were previously classified as “permanent-contact implant devices” (3,8), in vitro biocompatibility tests (cytotoxicity, tissue implantation assays, etc.) are required before advising wide range clinical use. Various studies have concluded that MTA can induce the regeneration of periodontal ligaments (9,10).

Osteoblasts also displayed a favorable response to MTA. Studies have also found the apposition of a cementum-like material and formation of bone (9,10). MTA consistently offered a biologically active substrate by stimulating interleukin production (3,11).

Under these circumstances it is very favorable for BA to be compared with MTA for its biocompatibility and cytotoxicity cause as mentioned before they are very similar in many ways but developed to serve as a better choice. The purpose of this study was to evaluate and compare the cytotoxic effect of bioaggregate and mineral trioxide aggregate on subcutaneous connective tissue of rats.

## Material and Methods

This study was revised and approved by the Istanbul University Local Committee on Animal Research Ethics. Fifty male Sprouge Dawley rats weighing 250-300 g were used in this study. All ethical criteria in care and use of laboratory animals were observed according to the Istanbul University Local Committee on Animal Research Laboratory (Vote number 10/2009). In this study the animals were divided into 3 groups.

Group 1: In this group ProRoot MTA (Dentsply, Tulsa Dental Products, Tulsa, OK) was used.

Group 2: In this group DiaRoot BA (DiaDent, Burnaby, BC, Canada) was used.

Group 3: Control group

All 3 groups were divided into 5 subgroups (n=10 rats) according to estimated time intervals of 7, 15, 30, 60, and 90 days. Both MTA and BA were prepared according to the manufacturers’ directions, and they were immediately placed in sterile polyethylene tubes with 1.0-mm internal diameter, 1.5 mm external diameter, and 10-mm length. Animals were anesthetized with 25 mg/kg of ketamine plus 10 mg/kg of xylazine, and their backs were shaved and disinfected with 10% Batikon (Kim-Pa, Istanbul, Turkey). Three 3-mm incisions with a number 15 blade (Aesculap, Tuttlingen, Germany) were made at least 3 cm apart on the back of each rat in a head-to-tail direction. The test materials were implanted into 2 separate incisions. The third received an empty tube as a control in each rat. The incisions were closed with a 3/0 silk suture. After 7, 15, 30, 60, and 90 days, the rats were sacrificed in an induction chamber by using a high dose of ketamine (Bayer Turk, Istanbul, Turkey). The tubes and surrounding tissues were excised in a 2-2 cm² dimension and fixed in 10% buffered formalin (Merck, Darmstadt, Germany) for 2 weeks. Sections of 5-µm thickness were made perpendicular and as near as possible to the opening of tubes and stained with hematoxylin and eosin to evaluate inflammatory reactions (12)and mineralized structures (dystrophic calcification) in the tissue. Microscopic evaluations were made through a light microscope (Carl Zeiss, Oberkachen, Germany) at 100 and 400 magnifications.

Evaluations of inflammatory cells were made in five separate areas (13).

The inflammatory reactions were scored as follows.

Grade 0: Zero or few inflammatory cells and no reaction,

Grade 1: Fewer than 25 inflammatory cells and mild reaction,

Grade 2: Between 25 and 125 inflammatory cells and moderate reaction,

Grade 3: 125 or more inflammatory cells and severe reaction (13).

Evaluation of fibrous capsules was scored in 2 groups:

1. thin (less than 150 mm),

2. thick (more than 150 mm) (13).

The quantity of dystrophic calcification was scored as follows:

3: more than two thirds of the tube periphery.

2: between one third and two thirds of the tube periphery.

1: less than one third of the tube periphery.

0: when no calcification existed (14).

In our histological evaluation we also included the presence of necrosis and foreign body reactions in the soft tissue around the implanted materials. Presence of necrosis was scored as follows:

1. Present

2. Not present

Foreign body reactions were scored as follows:

1. Present

2. Not present

Histological sections were evaluated by 2 independent operators who were blinded to the materials and time periods.

-Statistical analysis

Statistical calculations were performed with NCSS (Number Cruncher Statistical Systems) 2007 statistical software (Utah, USA) program for Windows. In addition to the standard descriptive statistical calculations (frequency, percent), chi-squared test and Fisher’s exact test were performed for the evaluation of the quantitative data. Statistical significance level was established at p<0.05.

## Results

-Inflammatory reactions

**Figure 1:**
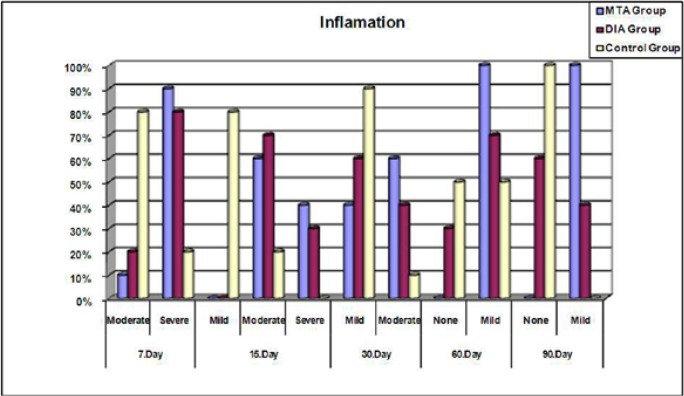
Inflammatory reactions of the tested materials at each evaluation periods (%).

In all groups a severe to moderate chronic inflammatory reaction was observed at 7 and 15 days, which decreased with time. Both the MTA and BA groups showed severe inflammatory reactions compared to the control group at 7 (p=0.005, p=0.023) and 15 days (p=0.002, p=0.001), but there was no statistically significant difference between the MTA and BA groups (p=0.999, p=0.639). The presence of an inflammatory reaction in the MTA group was significantly greater than in the control group at 60 and 90 days (p=0.032, p=0.0001). The BA group, by contrast, did not show any significant difference (p=0.303, p=0.087). The inflammatory reaction difference for both the MTA and BA groups was statistically significant at 7, 15, and 30 days. After 30 days a moderate to mild reaction was observed. At 60 days no statistically significant difference was observed between the MTA and BA groups, whereas at 90 days the MTA group showed a significantly greater reaction than the BA group (p>0.05) ( Table 1, Fig. 1).

**Table 1:**
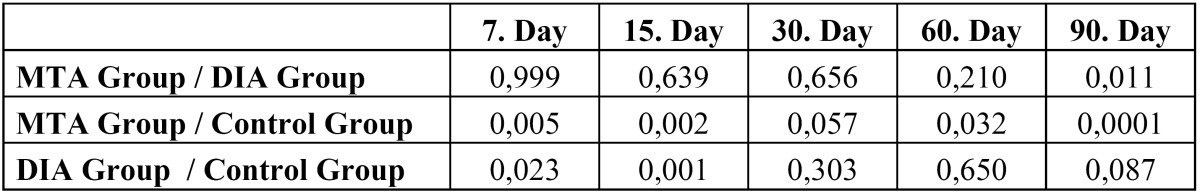
Comparison of inflammatory reactions of groups at each evaluation period.

-Evaluation of fibrous capsules

**Figure 2:**
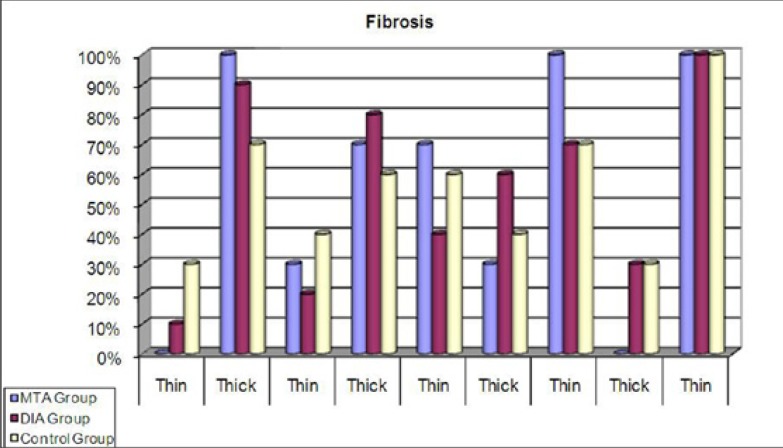
Fibrous capsules presence of the tested materials (%).

For all groups at all time intervals, the distribution of fibrous capsule (FC) presence was not statistically significant (p>0.05). In the MTA group the presence of FC at 7 days was significantly greater than at 30, 60, and 90 days (p=0.003, p=0.0001). At 15 days the presence of the capsules was greater than at 60 and 90 days (p=0.003). More FC were observed in the BA group at 7 days than at 60 and 90 days (p=0.02, p=0.0001). The observed FC at 15 and 30 days were greater than at 90 days (p=0.0007, p=0.011) ( Table 2, Fig. 2).

**Table 2:**
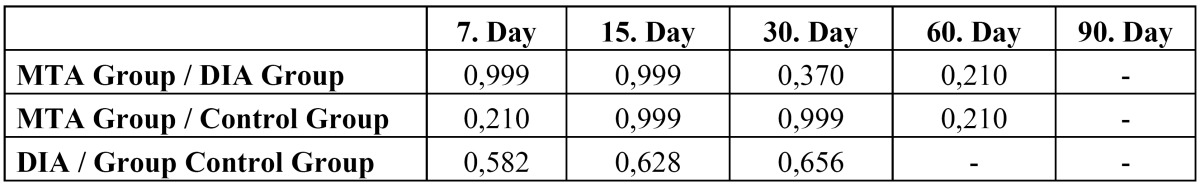
Comparison of fibrous capsules presence at each evaluation period.

The quantity of dystrophic calcification

**Figure 3:**
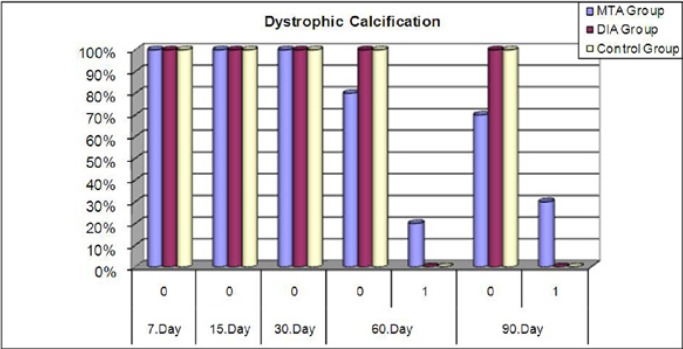
Dystrophic calcification of groups at each evaluation period (%).

At all time intervals for the BA and control groups, there were no dystrophic calcifications (DC) present. In the MTA group at all time intervals there were statistically significant differences in the distributions of DC (p=0.064) (Fig. 3).

-Presence of necrosis

**Figure 4:**
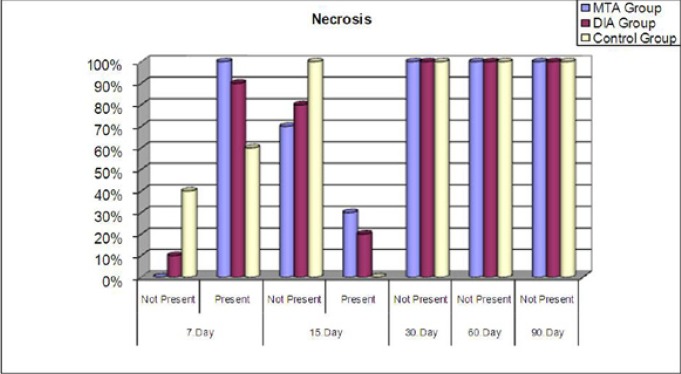
Necrosis presence of the tested materials at each evaluation period (%).

No necrosis was observed in any group at 30, 60, and 90 days. In the MTA group, necrosis was significantly greater at 7 days than at 15, 30, 60, and 90 days (p=0.003, p=0.0001). The BA group also showed statistically significant results at 7 days only (p=0.005, p=0.0001) (Fig. 4).

-Foreign body reactions

At 7 and 15 days foreign body (FB) reaction was observed in all groups. With similar distributions at 30 days, the MTA and BA groups presented significantly a greater FB reaction than the control group (p=0.003). At 60 and 90 days the MTA group showed a greater FB reaction than the BA and control groups, which was statistically significant (p=0.0001). In MTA group FB reaction was observed for all specimens at all days ( Table 3).

**Table 3:**
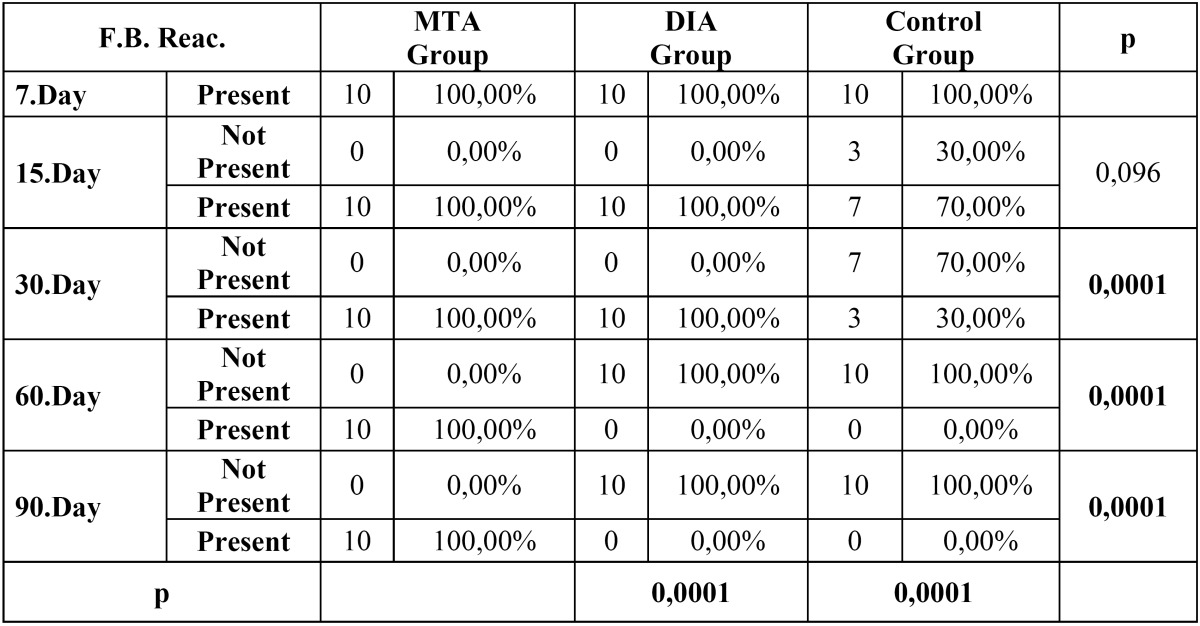
Foreign body reactions (F.B Reac.) of groups at each evaluation period.

## Discussion

Materials used in endodontics are frequently placed in close contact with the periodontium and thus must be nontoxic and biocompatible with the neighboring tissues. Bioactivity refers to the ability of a biomaterial to produce a chemical bond with vital tissues through a compliant interface (15). There are few data about BA’s biocompatibility. Therefore, the present study was designed to assess and compare the cytotoxic effects of MTA and BA (3).

The biocompatibility and induction characteristics of MTA are not completely understood and are likely multifactorial (16,17). Bioactivity is thought to be the reason behind the biocompatibility and mineralization induction capacity of MTA (17). Sarkar et al. (18) suggested that calcium ions released by MTA produce superficial and interfacial hydroxyapatite (HAp) precipitate in contact with dentin in the presence of phosphate-buffered saline (PBS). It is characterized as B-type carbonated apatite (19). Known as biologic apatite, carbonated apatite represents the mineral phase of hard tissue, which is more similar to bone apatite than pure Hap (16,20).

There are several in vitro and in vivo tests to evaluate the biocompatibility of dental materials. These include testing the general toxicity profile of potential materials in a cell culture, implantation tests, and usage tests in experimental animals according to accepted clinical protocols. A number of biocompatibility and mutagenicity studies have shown that MTA is a biocompatible material (4,21). The results of a meta-analysis of MTA biocompatibility showed that MTA is more biocompatible than Super EBA, IRM, and silver amalgam (22).

Many studies have compared the subcutaneous reaction of MTA to other materials such as amalgam, CH, Super EBA, various root canal sealers, IRM, ZOE, cold ceramic, and ethoxybenzoic acid (EBA) on experimental animals (4,12). One study compared the subcutaneous reactions of AMTA and Endo CPM sealer to Sealapex. Results showed that for a brief interval (7 days), both types of MTA caused mild to moderate reactions that decreased with time (23). Results on Day 30 were similar to the control, and at Day 60 results were similar to Sealapex. No significant difference was seen between the two types of MTA. Mineralization and granulations were observed for all the materials (23). Results of the present study echoed those of previous studies (12,23) in that there were similar reactions caused by MTA. At 7 and 15 days both BA and MTA caused severe to moderate inflammation which decreased with time. But the reactions caused by MTA decreased significantly slower than BA. Another study investigating the effects of Endo-CPM-Sealer (EGEO SRL, Buenos Aires, Argentina), Sealapex (Sybron Endo, Glendora, CA), and Angelus MTA (Angelus, Londrina, Brazil) on cell viability and cytokine (interleukin [IL]-1beta and IL-6) production by mouse fibroblasts concluded that none of the materials was considered cytotoxic in fibroblast culture. Endo-CPM-Sealer, Sealapex, and Angelus MTA did not inhibit the cell viability. Angelus MTA induced IL-1beta releasing significantly more than did the control (24). Ma et al. (25) evaluated the biocompatibility of 2 root-end filling materials, Endosequence Root Repair Material Putty (ERRM Putty) and Paste (ERRM Paste), and compared them to gray mineral trioxide aggregate (MTA). In this study, ERRM Putty and ERRM Paste displayed similar in vitro biocompatibility to MTA.

These studies showed that subcutaneous responses to MTA range from necrosis to dystrophic calcification. In addition, at first, MTA produces a moderate to severe subcutaneous response, which subsides at longer time intervals (4). Consistently with these findings, in the present study, MTA showed similar subcutaneous responses which decreased with time.

With technological characteristics and composition similar to white MTA, differing mostly in that it is aluminum-free, BA is the first nanoparticulate repair cement introduced to the dental market. It allegedly promotes cementogenesis and forms a hermetic seal inside the root canal system, but few published studies indicate its effectiveness (3,6). In a study of extracted human maxillary incisors, the researchers compared the cytotoxic effects of MTA and BA as a root-end filling material. No statistically significant differences between MTA and BioAggregate were found in the experimental periods. DiaRoot BioAggregate displayed in vitro compatibility similar to MTA (3). Contrary to this study results, in the present study BA showed statistically significantly better results than MTA for biocompatibility. This could be due to laboratory animals (Sprouge Dawley rats) were used in the present study instead of in vitro test, thus in this study model BA demonstrated statistically significant biocompatibility compared to MTA. Yuan et al. (26) investigated the cytotoxicity of BA and the effect of BA on mineral-associated gene expression in os-teoblast cells, which is in contrast with our results. In our study, we found that MTA induced dystrophic calcification, where BA failed to show any dystrophic calcification. They suggested that BA appears to be a novel nontoxic root-end filling biomaterial and is able to induce mineralization-associated gene expression in osteoblast cells. In a study of iRoot SP root canal filling mate-rial, AH Plus and MTA were evaluated for their biocompatibility with L929 mouse fibroblasts. It was concluded that AH Plus root canal sealer was significantly more toxic to L-929 cells than MTA and iRoot SP. iRoot SP had intermediate toxicity (27). Mukhtar-Fayyad (28) evaluated and compared the cytotoxicity of 2 bioceramic-based materials, BioAggregate and iRoot, on human fibroblast MRC-5 cells. The researcher found that both BioAggregate and iRoot SP displayed an acceptable biocompatibility. Also, the cytotoxic effect of both materials was concentration-dependant.

## Conclusions

Based on the results of this study, we can conclude that both BA and MTA are biocompatible materials. However, the BA group showed significantly better inflammatory and foreign body reaction than the MTA group. Therefore we suggest that BA is more biocompatible than MTA. However, MTA showed better results at presence of dystrophic calcification compared to BA.
